# Design and implementation of a low cost bio-printer modification, allowing for switching between plastic and gel extrusion

**DOI:** 10.1016/j.ohx.2021.e00186

**Published:** 2021-02-26

**Authors:** Adolf Krige, Jakub Haluška, Ulrika Rova, Paul Christakopoulos

**Affiliations:** Biochemical Process Engineering, Division of Chemical Engineering, Department of Civil, Environmental and Natural Resources Engineering, Luleå University of Technology, SE-971 87 Luleå, Sweden

**Keywords:** Bioprinter, FDM, 3D printer, Gel extruder

## Abstract

Due to the high cost of bioprinters they are not feasible for proof of concept experiments or educational purposes. Furthermore, the more affordable DIY methods all disable the plastic printing capability of the original printer. Here we present an affordable bio-printing modification that is easy to install and maintains the original capabilities of the printer. The modification used mostly 3D printed parts and is based on the popular, open-source Prusa i3 3D printer. The modifications are kept as simple as possible and uses standard slicing software, allowing for installation by less experienced builders. By using disposable syringes and easily sterilizable parts, an aseptic bioprinting setup can be achieved, depending on the environment. It also allows for 2 component printing as well as UV curing. The bio-printing and curing capabilities were shown by printing and curing an artificial biofilm of an electro-active bacteria, Geobacter sulfurreducens, onto a carbon-cloth electrode which was used in a microbial fuel cell.

## Graphical abstract

**Figure:**
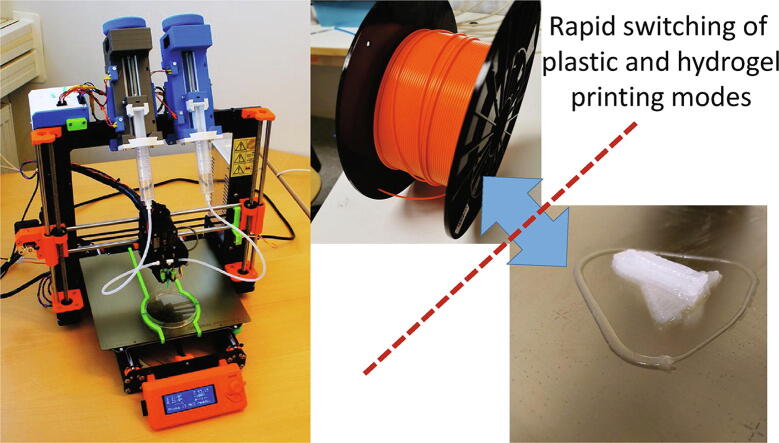

Specifications table

**Table:** 

Hardware name	*Please specify the name of the hardware that you invented/customized*
Subject area	• Engineering and Material Science • Medical • Biological Sciences • Educational Tools and Open Source Alternatives to Existing Infrastructure
Hardware type	• Mechanical engineering and materials science
Open Source License	CC BY-SA 4.0
Cost of Hardware	*Approx.* 300 *USD for a two extruder system*
Source File Repository	https://data.mendeley.com/datasets/3kfmsz4n28/1

## 1 Hardware in context

In the field of 3D gel-printing there are several proprietary 3D bio-printers, which are usually just one-task (gel-printing) machines, incapable of performing other tasks. The price of these type of systems have fallen significantly recently, with prices starting from around 5 000 USD for the most basic system and going up to several hundred thousand USD for more advanced systems [1]. However, 5000 USD is still expensive for a single purpose educational tool, or even for proof of concept research experiments.

There have also been developments in open source DIY 3D printers and extruders. These are typically designed on a case-by-case basis, for specific purposes [2], [3]. The printers are usually based on Rep-Rap fused deposition modelling (FDM) 3D printers. However, a huge disadvantage of these systems is that, when the printers are rebuilt as a gel printer, the original 3D plastic printing is disabled, effectively resulting in the loss of a 3D printer. Switching the printer back to enable the printing of plastic also requires tools and significant time. Two examples of this type of modification is the large volume extruder [4], Replistruder [5]. Both are used for FRESH 3D bio-printing. Another option is to build a printer from scratch, allowing you to choose your desired features, such as heated/cooled bed, sensors, enclosure, build volume etc. However this requires a significant amount of knowledge and skills [6].

An FDM 3D printer is an extremely useful tool in a lab or classroom, for printing specialized equipment. A solution was therefore needed to keep the original functionality of the printer. The main goal of this project was to create an open-source device which is easy to assemble and operate and can be used for the 3D bio-printing of hydrogels as well as for FDM printing. Our solution is a DIY 3D bio-printer modification that allows one to print melted plastic as well as biogels without having to rebuild the printer inbetween. The 3D printer maintains all its features and the add-on device is connected to the printer without limiting the original functionality of the printer significantly. The add-on device consists of a gel extruder (up to 2 extruders) and additional control device, which controls the gel extruders and all of the end stops. This implementation makes the system somewhat more expensive (approx. 300 USD) than some other applications (such as the ultra-low cost bioprinter [7]) since it requires the use of additional stepper motors and an Arduino. However, since the FDM functionality is maintained a second printer is not required, essentially saving the cost of a second FDM printer. In addition, it allows for the printing of 2 bio-ink materials as well as allowing for UV-curing.

Although the main aim of the system is simply to allow for bio-printing while maintaining the FDM functionality, the development of specialised FDM filaments (such as dissolvable or conductive materials) allows for an extreme diversity in possible applications. Some possible examples such as the printing of bio-sensors (for example a bacteria-based AND logic gate [8]) connected with conductive FDM filaments, or the ability to print single use reactor bases into which can be bio-printed onto. However a detailed summary of possible applications is outside the scope of this work.

## 2 Hardware description.

The modification was designed to serve as an entry-level tool for researchers or educators, allowing one to experiment with gel-printing without disabling the FDM ability of the 3D printer.

The printer conversion was designed to change as little as possible on an original Prusa i3 MK3 3D-printer. The Prusa I3 MK3 3D printer was chosen as the base for the 3D bio-printer because it is a well-engineered, inexpensive (750 EUR, or less if a replica is used) and very common 3D printer. The printer is also open source, so it is easy to modify both the hardware and the software of the printer.

The project was focused on the implementation of the following additional functions to the original printer:

• Two material gel printing with automated material switching.
• Controlled UV LED Curing
• Easy and aseptic bio-gel exchange
• Automated print abort when the biogel is finished, to prevent damage

**Fig. 1:**
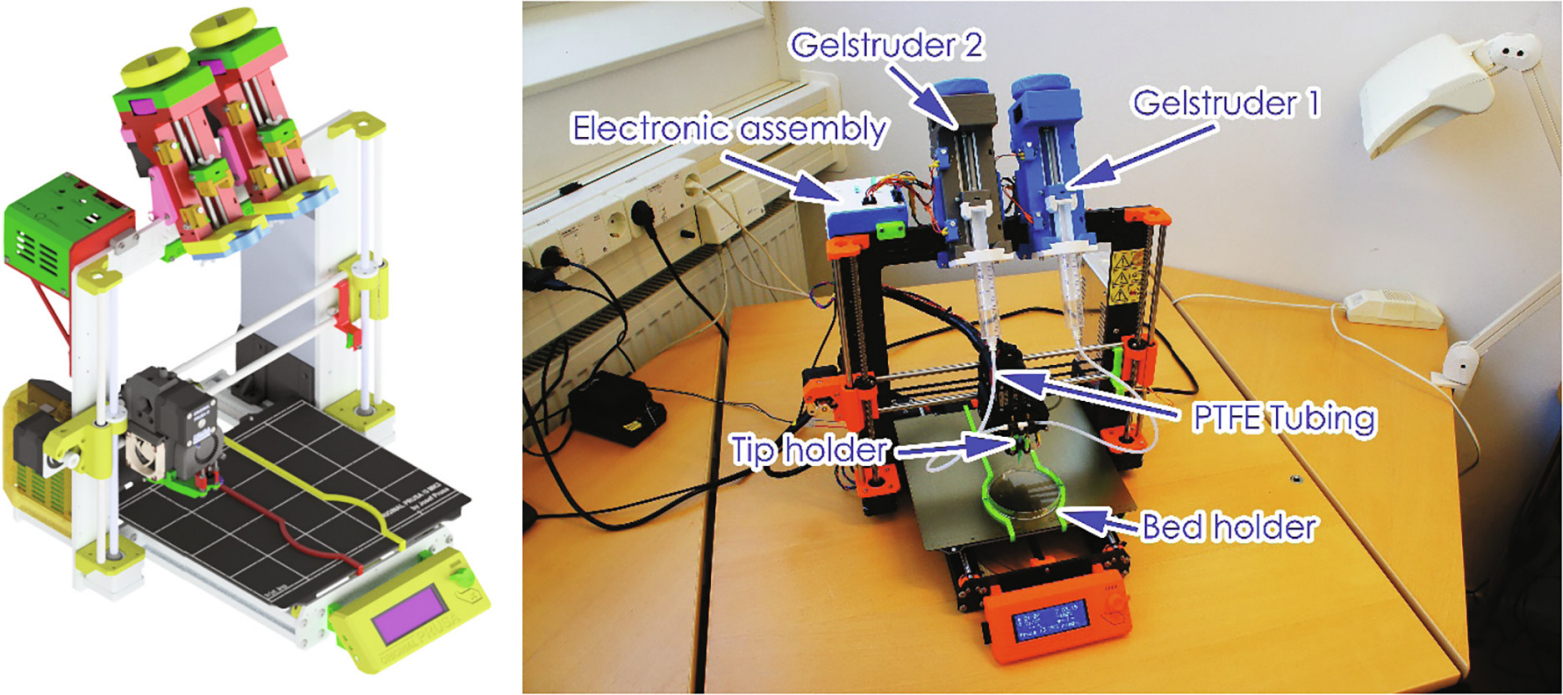
Gel printer model, and final assembly.

An Arduino board control system was added in order to control the extruders and limit switches (on both the extruder and printer), and to manage switching between the two Gelstruders. A box was added for the additional electronics and placed on the printer frame. This box contains the Arduino UNO board, relay shield and a shield with connectors and other electronic components. A simple manual switch allows the user to change from the original FDM printing to the gel-printing mode, this should only be used when the stepper motors are not powered. The assembled printer-modification, with gel extruders and electronic is shown in the Fig. 1. The gel-capable extruder (Gelstruder) was designed to be attached to the top of the printer frame, with a PTFE tube connected to the printer carriage. The design of the extruder is based on the large volume extruder [4] and the Replistruder [9] and built from 3D printed parts and parts that can be purchased from a standard hardware store.

**Fig. 2:**
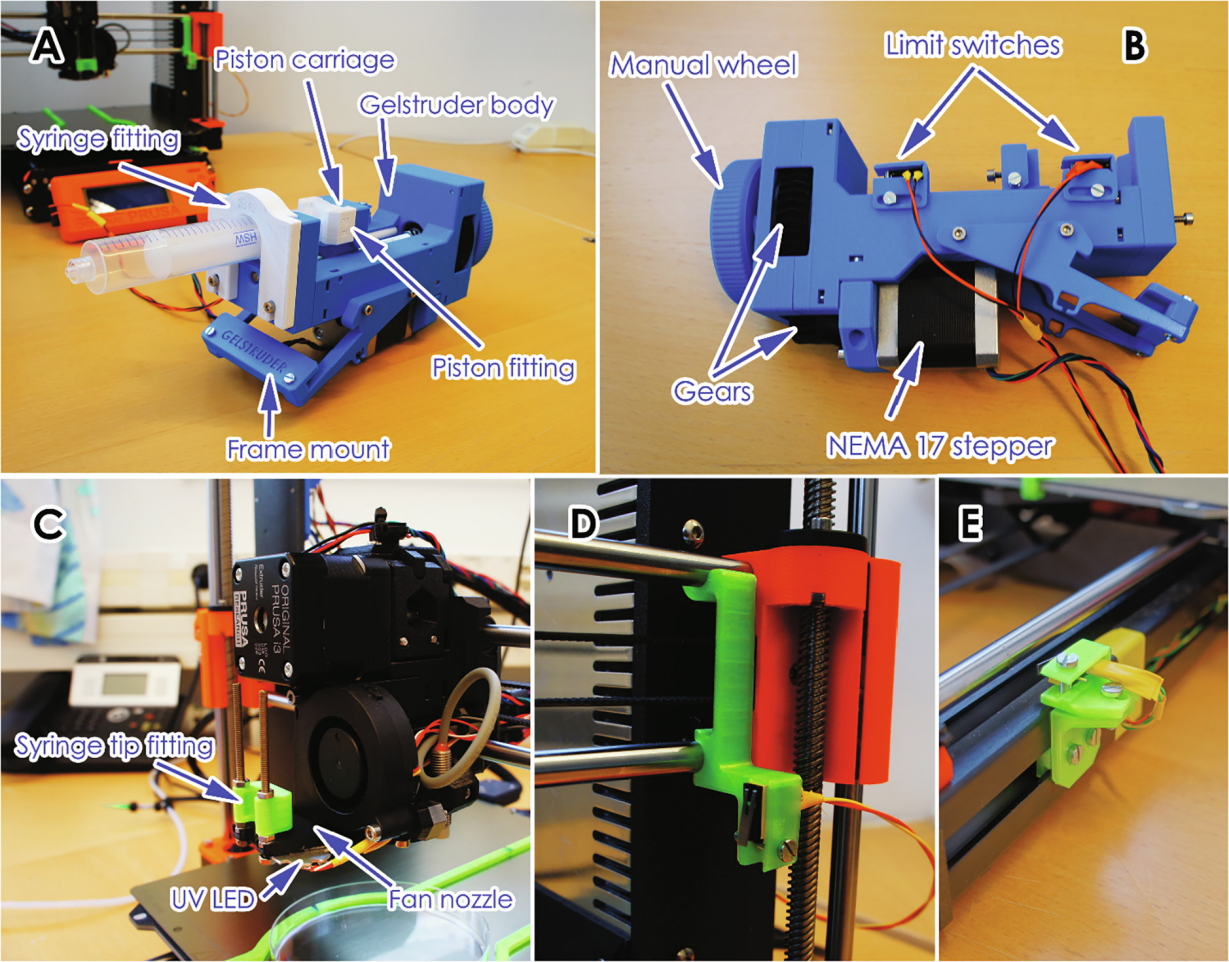
Detailed design images showing switch placements.

A 10/20 ml syringe was fitted into the gel extruder (Fig. 2 A), the syringe is connected to a PTFE tube and a nozzle is connected to the tube via Luer-lock fittings. Different syringe sizes (such as 5 ml) can also be used by modifying the 3D printed syringe fittings (white fittings in Fig. 2 A), yet due to the dead volume in the system a syringe below 5 ml is not recommended. However, for more expensive material or smaller print sizes, the system or syringes can be manually primed with a sacrificial material. This can be done by first filling the syringe partway with a low-cost hydrogel followed by the intended bio-ink.

The extruder was also fitted with adjustable limit switches to stop the extruder and prevent it from damaging itself (Fig. 2 B). A blunt tip plastic/stainless steel needle is used as the printing nozzle (an 18G needle diameter is used in the examples) and the nozzle was attached to the side of the print head using a snap-in clip. The UV LED (365 nm, MTSM365UV-D5120S, Marktech) was glued to the bottom of the print head as close to the syringe tip as possible (The metal heat plate of the LED can be seen in Fig. 2 C). The UV LED is switched on/off via an optocoupler and powered via a LED driver, which also allows for dimming using pulse-width modulation. The UV LED can be triggered either at the end of the print or between each layer, using custom G-code.

**Fig. 3:**
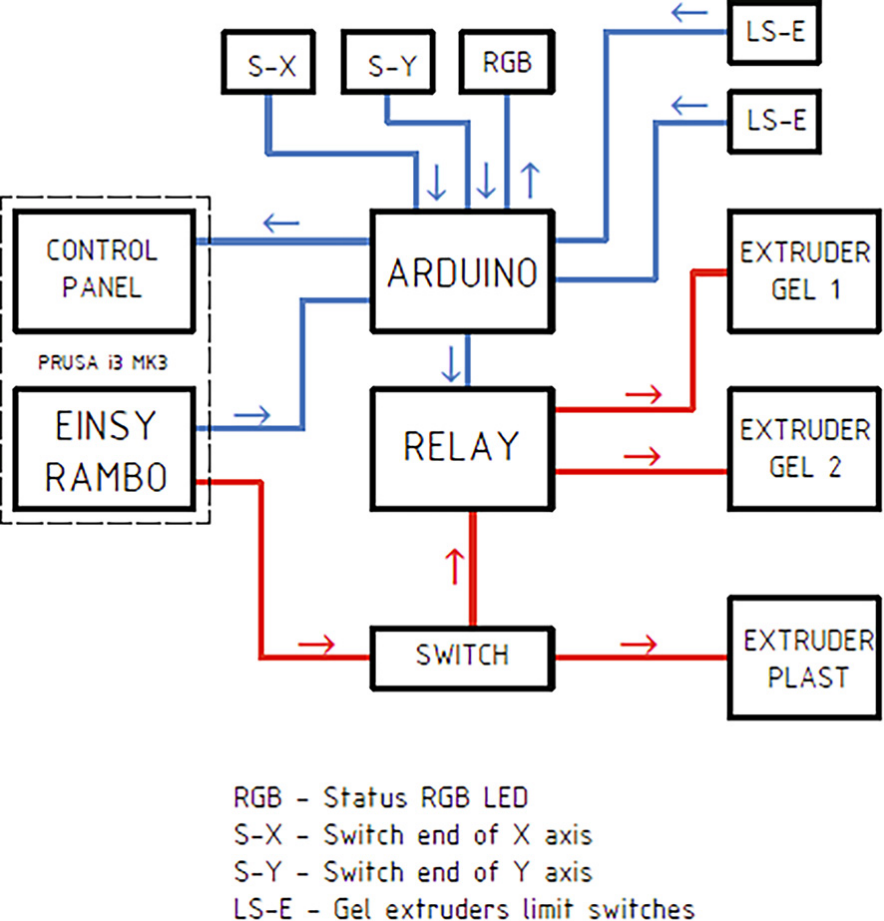
Diagram of the Printer and gel Extruders control setup. The blue lines represent data lines, with the arrow signalizing the flow of the data. The red lines are the power lines of the stepper motors. (For interpretation of the references to colour in this figure legend, the reader is referred to the web version of this article.) (For interpretation of the references to colour in this figure legend, the reader is referred to the web version of this article.)

The external Arduino board controls the UV LED and the relay shield, which is used for switching between the two gel extruders. A schematic of the control implementation is shown in Fig. 3. The blue lines represent the data lines and the arrow the flow of information, while the red lines represent the power lines of the stepper motors. The Arduino is only connected to one unused pin in the printer mother board (Einsy board) and wired to the control switch of the printer, allowing for output and input to the printer. The Arduino monitors the signal from the extruders’ limit switches and the additional switches at the end of the printer axis. An RGB LED light was added to show the status of the Arduino board. The extruders are wired to a relay, which is wired in turn to the printer’s extruder control through a 3-way switch. The physical switch is used to switch between plastic and gel printing, while the Arduino board can switch between two Gelstruders via the relay board, allowing for a multi-material print.

Three “trigger” methods were included in the system, i.e. two limit switches at the far-end of the printer’s X and Y axes (which are pressed by moving the print head to the far edges using custom G-code), as well as a direct connection to an unused pin in the printer’s motherboard (pin 76). The limit switches are placed at the opposite side of the home position of the printer, so that they are not triggered when homing (Fig. 2 D&E). This allows for three lines of communication between the printer and the Arduino board without the need for complex changes to the printer’s firmware. These can be assigned in the Arduino code to trigger different functionalities based on the requirements of the print. Changing the function of each trigger does not require any physical changes to the setup, since this is assigned in the Arduino code and G-code. In this case they were assigned as follows:

• Y-axis switch: Switch the UV LED on/off
• X-axis switch: Switch between the two gel extruders
• Pin76: Monitor the printer status and start the Arduino program

The Arduino program is initially in a “ready mode” waiting for a signal from the P76 pin. This allows the limit switches on the extruders, the UV-LED and the extruder relay to be inactive while not printing. This prevents the auto stop from automatically triggering if you accidentally touch a limit switch as well as keeping the UV-LED from going on while not wearing safety glasses or the relay from switching stepper motors while moving (under load).

Furthermore, since the printer’s native extruder control is used this allows for the additional functionality without extensive firmware changes. Because of this, the same freely available software (Sli3r PE) can be used for data preparation, for both the plastic and the gel printing. The change only requires a custom profile for gel-printing, with specific routines (files given in Mendeley dataset). Other parameters need to be adjusted to the specific gel or setup being used. For example, parameters such as a layer height and width are adjusted in order to work with different needle sizes. This profile can also be simply edited according to the application of material characteristics. Once the profile is created the software should work for different models without additional changes. The user can control the relative gel flow rate as well as the UV LED and extruder switching by creating custom G-code routines.

For example, to set the relative flow rate of the gel, the extruder setting of “step per mm” is changed (Based on the use of a M5 screw rod transition) using the “M92 command”: M92 E2800 [10]. The original value for plastic print is E280. This can be fine-tuned based on the material properties and the needle size.

The modification was designed to be affordable as well as relatively easy to install and simple to use. This allows the setup to be used for a wide variety of applications:

• The affordability and ease of use allows the modification to be used as an educational tool. Giving students the opportunity to experience and play around with bioprinting, which might otherwise be impossible due to limited funds.
• The modification can also be useful for proof of concept prototyping, in order to evaluate project viability before applying for funding. The system also allows for different functionalities to be added based on the specific requirements.
• The printer can also be used to print other gels, such as sealants.

## 3 Design files

All 3D models, images of PCB boards, printer profiles with G-code and detailed instruction manuals is given in Mendeley data (https://data.mendeley.com/datasets/3kfmsz4n28/1)

**Design Files Summary** *[Please include a summary of all design files for your hardware by filling rows of the table below]*

**Table:** 

Design file name	File type	Open source license	Location of the file
*3D model files*	*.stl & .stp*	CC BY-SA 4.0	*DOI: https://doi.org//10.17632/3kfmsz4n28.1*
Arduino programming	.ino	CC BY-SA 4.0	*DOI: https://doi.org//10.17632/3kfmsz4n28.1*
PCB files	.jpg/.fzz	CC BY-SA 4.0	*DOI: https://doi.org//10.17632/3kfmsz4n28.2*
Custom print profiles with G-codes	.ini	CC BY-SA 4.0	*DOI: https://doi.org//10.17632/3kfmsz4n28.1*

*Design files: Includes the CAD files for the various 3D printed components of the modification.*

Arduino code: Includes the Arduino code used to control the gel extrusion.

PCB files: Includes the design of the Arduino shield containing the LEDs and relay connections

Custom G-codes: Includes an example of the custom G-codes used.

## 4 Bill of materials

The estimated total cost of the modification, including two extruders, is approximately 2900 SEK, or Approx. 300 USD (excluding the cost of the printer). However, the exact price would depend heavily on the price of parts at your local supplier. The price would also be approximately half as much if only one extruder is required, since only one stepper-motor is required and a relay shield is not needed. A complete bill of materials can be found at https://data.mendeley.com/datasets/3kfmsz4n28/1:

## 5 Build instructions

### 5.1 Gelstruder assembly

All the parts were printed using PLA. The only exceptions being the gears, which were printed using PETG. The only part that requires supports while printing is the main gear, the rest can be printed without supports using 50% infill and 2–3 perimeters.

**Fig. 4:**
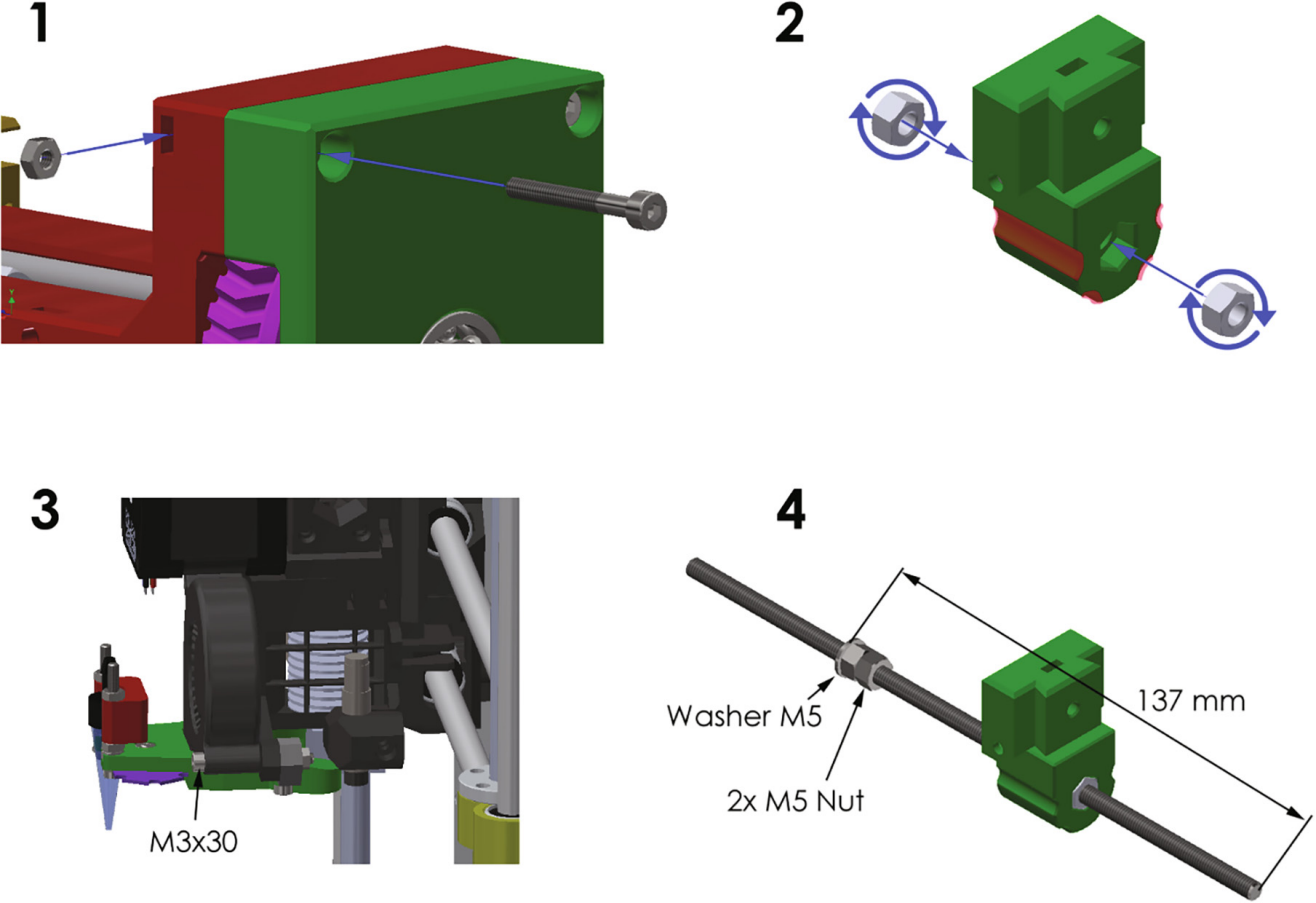
Additional assembly instructions.

The assembly of the hardware parts should be done according to the technical drawings in the attached files. A few additional instructions are presented below and in Fig. 4.

1. In the assembly a slot nut technique is used, the technique is presented below.
2. The play between the slider and screw rod can be reduced by turning the nuts in the slots. Furthermore, the highlighted areas of the slider should be sanded down to reach a good fit on the guide rods.
3. Replace the bolt at the left side of the fan nozzle with a M3x30 bolt.
4. Screw the M5 counter nut and the washer onto the screw rod at a distance of 137 mm.

### 5.2 Changing the firmware

**Fig. 5:**
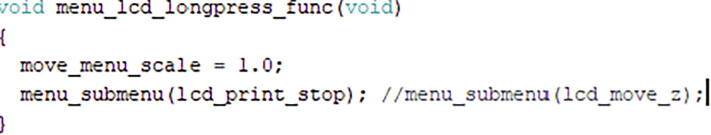
Changes in the firmware of the printer.

The firmware of the printer was changed in order to be able stop print if the limit-switch on the Gelstruder is engaged. There is only one change, shown in Fig. 5. The function of the “long press knob” is changed from the “move z axis” function to the “stop print” function in the “ultralcd.ccp”. Since this is a minor change and quick to implement, it should be relatively easy to keep with the firmware up to date, even though the firmware updates should not be necessary. If the firmware needs to be updated, please follow the manual at the following link: https://github.com/prusa3d/Prusa-Firmware.

From this link the user should download the Firmware folder, and then follow the steps for Windows build – Using Arduino. The modified firmware can be uploaded to the printer via Sli3r as a regular firmware update.

### 5.3 Electrical connections

**Fig. 6:**
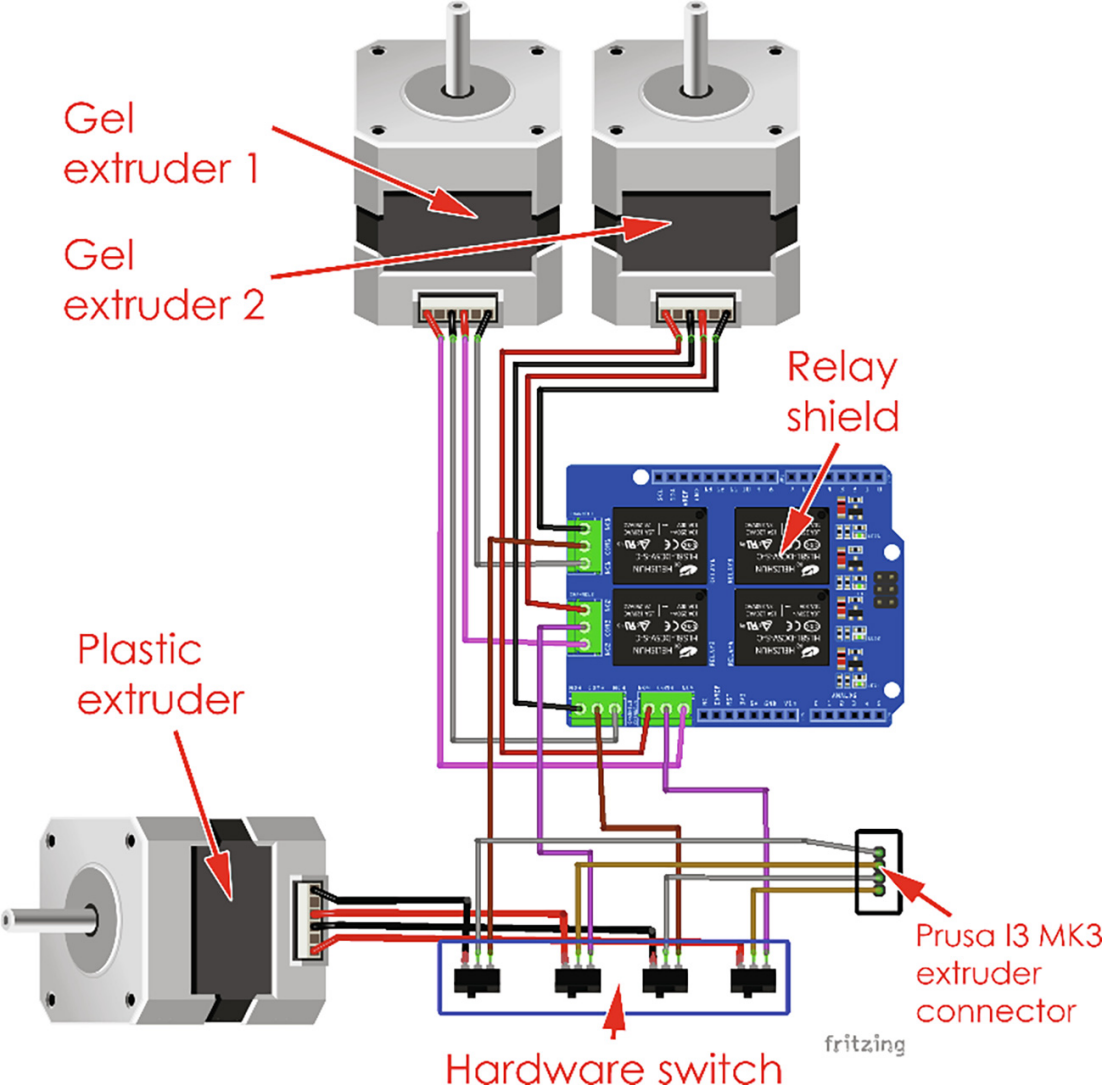
Extruder connection schematic.

**Fig. 7:**
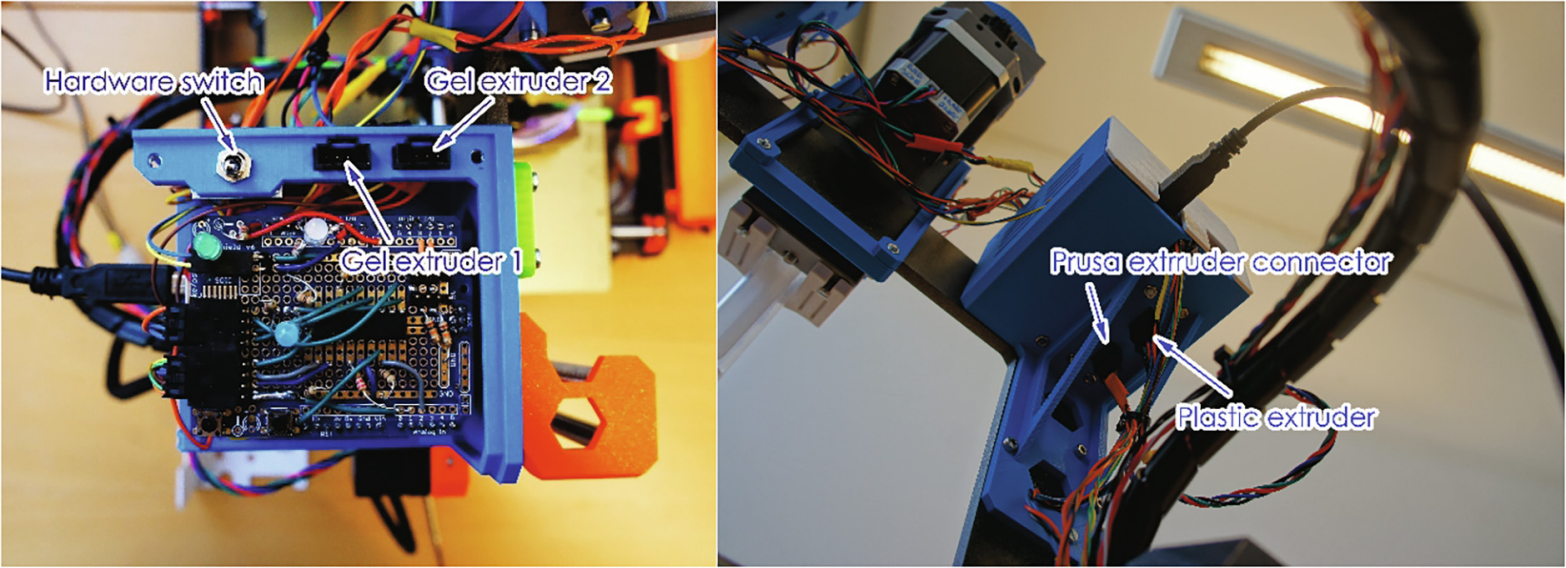
Control system wiring.

The Arduino assembly uses two stackable shields, first the relay shield and then the prototype shield that contains LEDs. The extruder connector of the Prusa motherboard is connected to a 4-chanel hardware switch, the output of which is connected to the relay shield and the original plastic extruder. This allows for a physical switch between the plastic and gel extruders. The relay shield is then connected to the two gel extruders, to enable electronic switching of the extruders. The extruder setup is shown in Fig. 6 and the overall wiring is shown in Fig. 7.

**Fig. 8:**
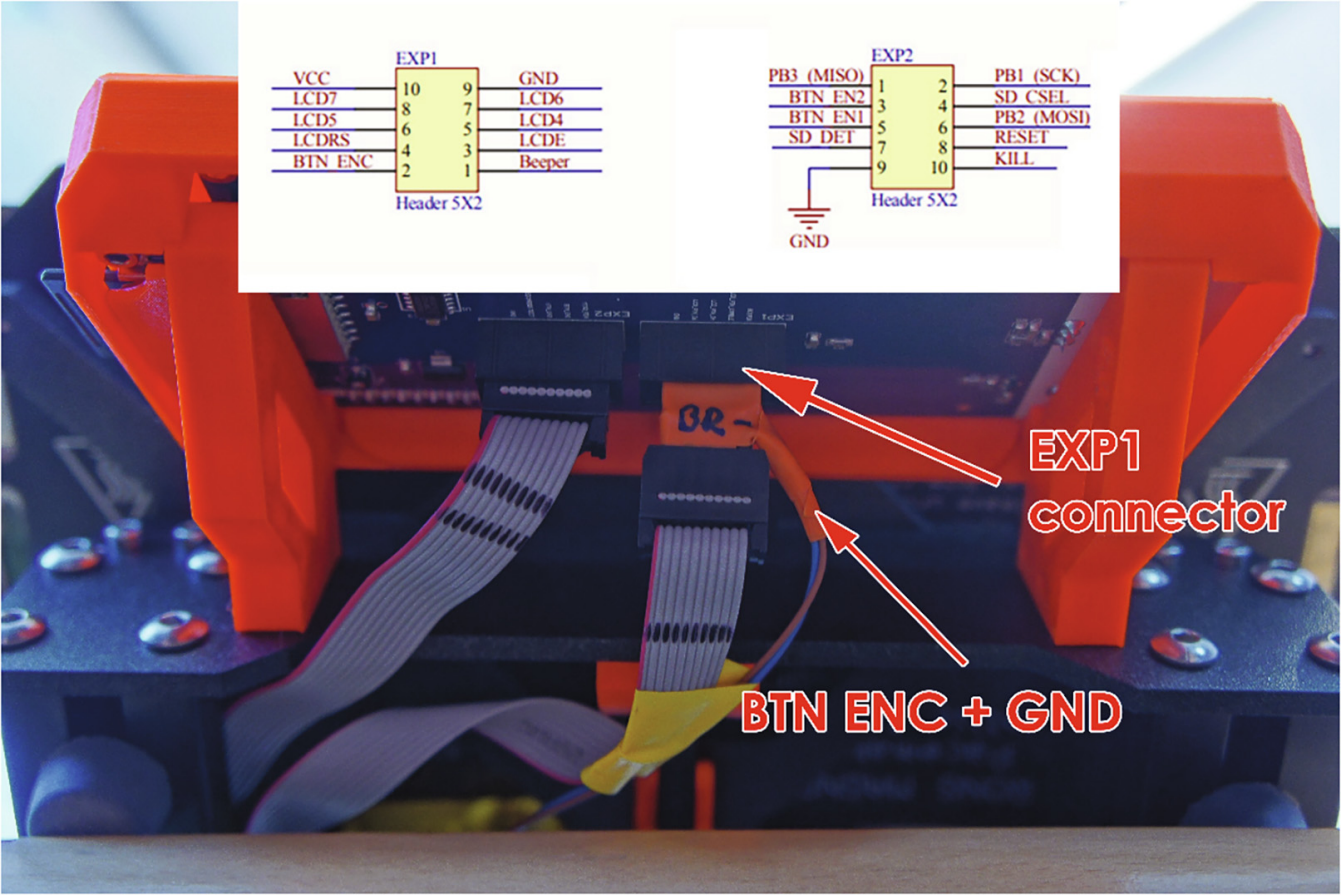
Control panel wiring modification.

The control panel of the printer is connected to the Arduino board as shown in Fig. 8. Where a connector is added and additional wires from this connector are connected to pins 5, 6 on the prototype shield. This allows the control unit to electronically abort a print when an extruder end stop has been reached, by simulating a “long press” of the knob on the Printer’s control panel. The simulation is done with connection of the knob signal and ground wires at the control panel. This does not affect the plastic 3D printing in any way, moreover it allows the user to stop the print quickly by simply long pressing the printer knob if anything goes wrong.

Finally, pin P76 and the ground pin in the same socket of the Prusa motherboard are connected to the pins 2,3 on the prototype shield. All the connectors for the additional switches and limit switches as well as a connector for a UV LED are soldered onto the prototype shield. The Arduino is powered from a separate power source, so it can be disabled while 3D printing plastic.

**Fig. 9:**
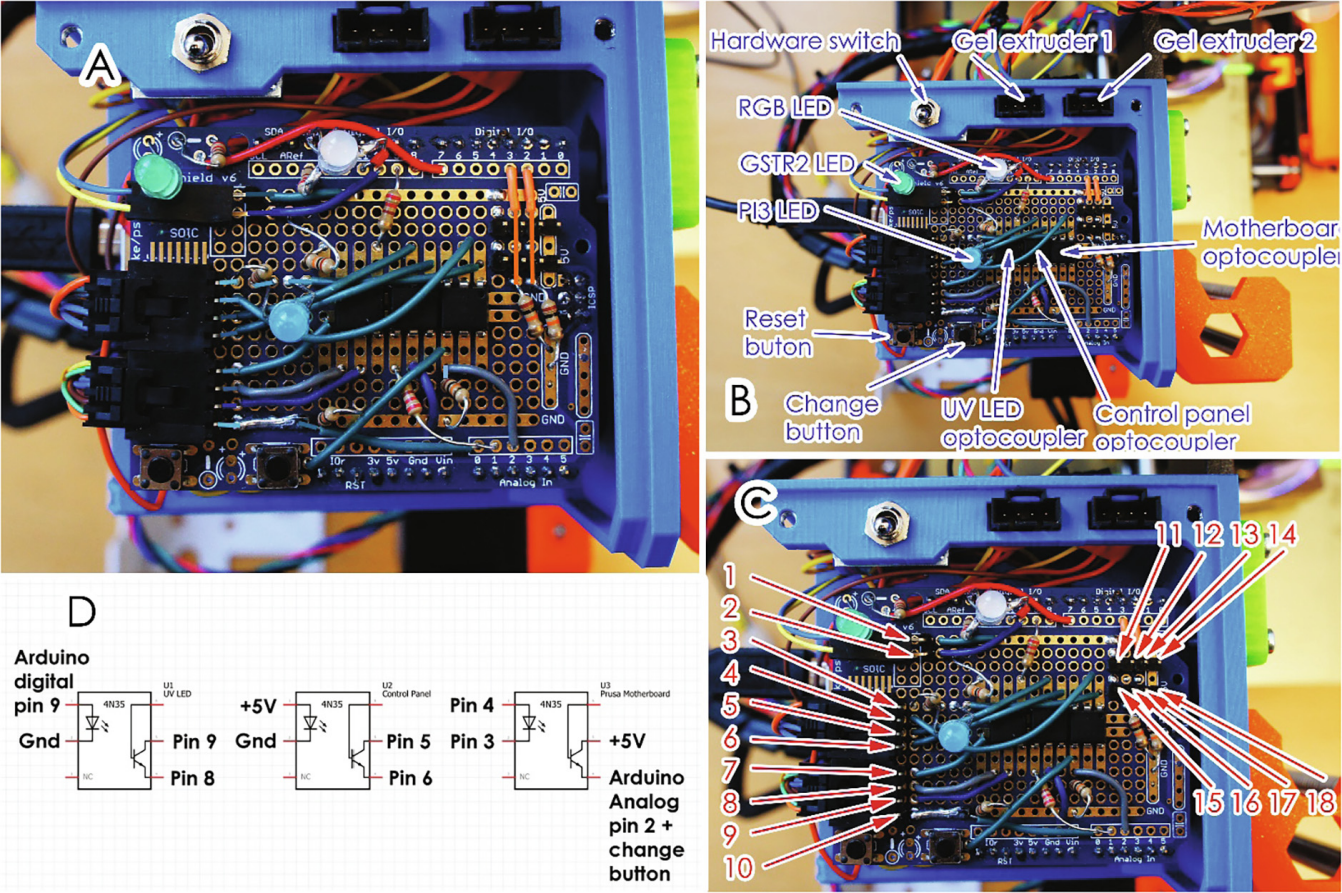
Prototype shield description.

The new control unit reads the voltage from the pin P76 on the Einsy Rambo circuit board of the printer. This pin is unused and is accessible via G-code while printing using G code M42 P76 S0/S255. The wiring of the prototype shield is shown in Fig. 9, A, B and C. A schematic of the optocouplers’ connections are show in the Fig. 9, D. The functionality and connection of the pins are listed in Table 1. By using optocouplers, the circuits of the Einsy motherboard and the new Arduino board are electrically isolated.

**Table 1:** Prototype shield pin connections.

Pin	Function
1	X- axis limit-switch (Gelstruder); +5V
2	X- axis limit-switch (Gelstruder) ground; signal
3	Prusa motherboard ground pin
4	Prusa motherboard pin 76
5	Prusa knob signal pin
6	Prusa knob ground pin
7	Y- axis limit-switch (UV LED) switch; +5V
8	UV LED driver ground pin
9	UV LED remote control pin (+12 V)
10	Y- axis limit-switch (UV LED) ground; signal
11, 15	Gelstruder limit-switch; +5V
12, 16	Gelstruder limit-switch ground; signal
13, 17	Gelstruder limit-switch ground; signal
14, 18	Gelstruder limit-switch; +5V

A UV LED used for photo-curing is powered by separate power source via an UV LED driver, which is also controlled by the Arduino board. The control unit also contains a status LED indicator. The meaning of each colour used is explained in Table 2.

**Table 2:** Status LEDs meaning.

LED		Explanation
Pi3 LED		Indicates that the printer is turned on. This LED is on even if the Arduino board is not.
Gelstruder 2 LED		When ON it Indicates that Gelstruder number 2 is selected
Status RGB LED	RED	“Total stop” Indicates that the printer reached a limit switch and the print is aborted. After which that the Arduino board goes to the “Ready” mode
	GREEN	“Ready mode” Indicate that the Arduino is in the ready mode – no switches are active here.
	BLUE	“Printing” Indicate that the Arduino is in the printing mode – switches are active and the print is going.

### 5.4 Optimizing extruder placement and PTFE length

**Fig. 10:**
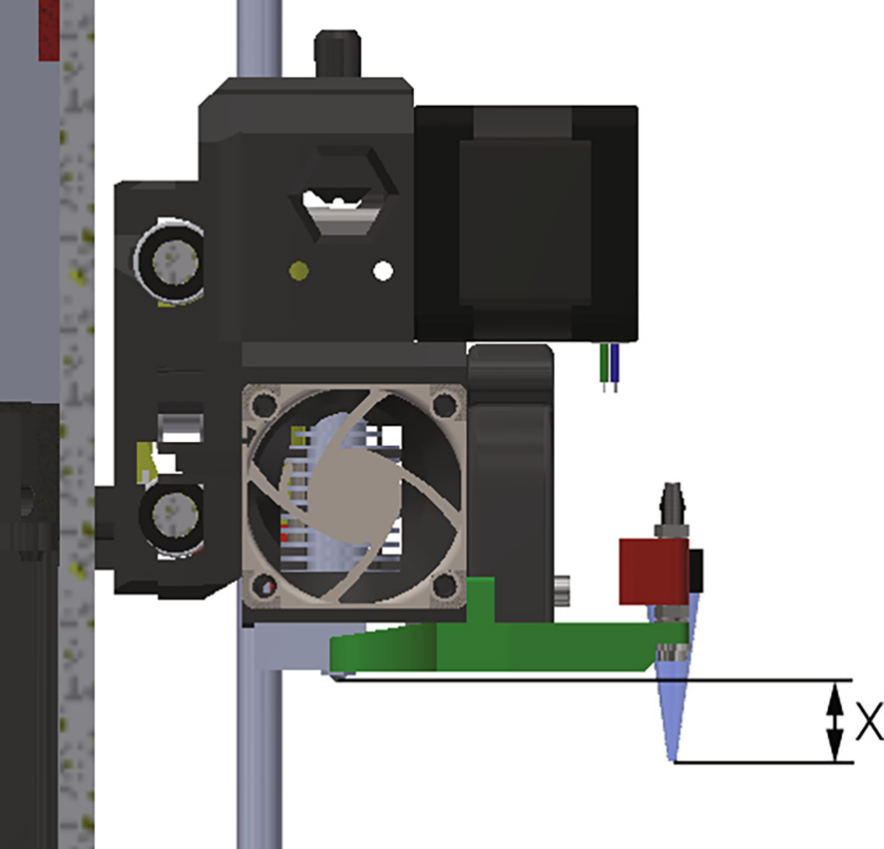
The Z offset measurement.

To minimize the pressure increase and dead volume caused by the PTFE tube, the PTFE tube needs to be as short as possible. The position where the print head is furthest from the extruder is typically when the print head re-calibrates the z-height at the start of the print, causing the syringe tip to drop below the bed level (The tip holder is designed so that the tip will hang over the edge of the bed). To calculate the minimum tube length required move the print head as far left and as far down as possible, with the plastic extruding nozzle almost touching the bed. Once that is done, install the desired syringe size and connect the PTFE tube to the syringe and a tip installed in the print head. Finally, cut the PTFE tube to the minimum length that will reach without putting stress on the printing syringe tip. If desired, the tube length can be further shortened (by the Z offset measurement seen in Fig. 10) if the automatic z-height calibration is skipped in the G-code. This method will however require a manual z-height calibration with every print, which can be tedious.

## 6 Operation instructions

### 6.1 Generating G-code

The routine of using Sli3r software for gel prints is the same as standard plastic printing, with only some parameters that need to be changed, as well as adding custom G-codes to the printing profile. Before slicing the model, the user should test and tweak the optimal parameters for the printing material. The attached G codes (https://data.mendeley.com/datasets/3kfmsz4n28/1) are optimized for printing a 2% Hyaluronic acid hydrogel.

An important value to set, each time the setup is changed, is the Z offset parameter. This is the difference in height between the tip of the original printer nozzle and the gel-printing tip, as shown in the Fig. 10. If the print is going to be performed on an object, the height of this object should be added to the Z offset parameter.

The printer in gel-printing mode uses only one reference point, near the edge of the printing surface, when homing the Z axis, and the automatic bed-levelling is disabled. Therefore, when the Z-axis is calibrated, the extruder tip hangs over the side of the printing surface.

### 6.2 Custom G-codes

Via the custom G-code sections in the printer settings section of the slicer software, it is possible to control the UV LED, switch the extruders and switch the Arduino board to printing mode. This is done by moving the print head to press one of the switches at the far ends of the X&Y axis, which triggers the command in the Arduino to switch on the UV-LED or to switch between the two gel extruders. Additional G-code can then be used, after moving to the switch, to move the UV-LED over the print for the desired duration. The duration is set by changing the path and speed that the print head takes, before returning to switch off the UV-LED again. These changes are added automatically to the G-code file when sliced using this profile. An example of the custom G codes used can be found in the attached dataset. The custom G-codes for Gel-printing are annotated and should be relatively easy to follow and modify.

### 6.3 Loading the syringe

**Fig. 11:**
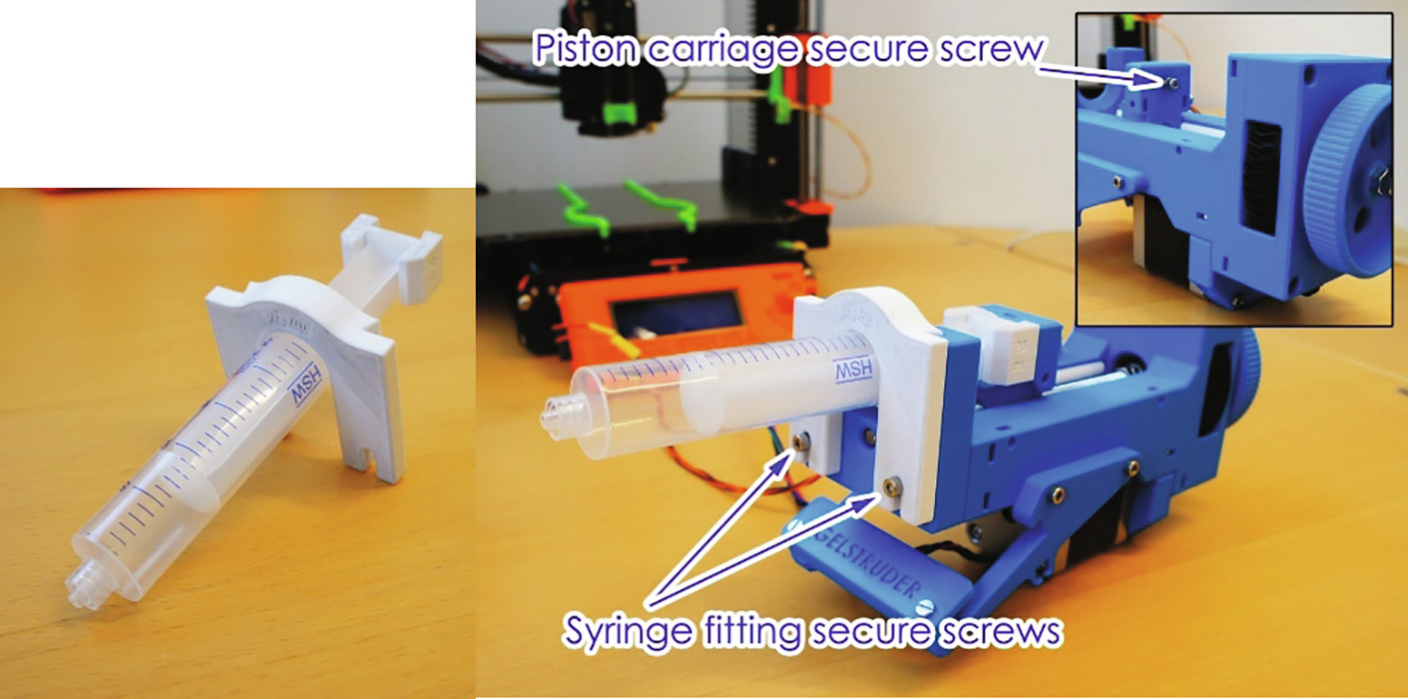
Syringe in the holder and Gelstruder securing screws.

To load the syringe first remove the air and prime the printing tube. The syringe is then placed in the syringe fitting as shown in Fig. 11. The syringe is the fitted either manually, by turning the manual wheel until the syringe holder slides into place or by using the G-code “Gelstruder-loading.gcode” as if printing a file. This is useful if the piston holder is far from the desired location. The G-code loading procedure is then as follow.

1. Run the G-code and make sure that the status LED is in printing mode (blue). If not, use the change button to change it.
2. Wait until the printer completes the “home Z” procedure.
3. The Gelstruder 1 is always selected by default at the start of a print (due to the wiring in the relay shield, described above). In case Gelstruder2 is used, the change is done by pressing the switch at the end of the X-axis (Fig. 2, D) by hand. After selecting the Gelstruder, the printer knob is pressed to continue.
4. The selected Gelstruder will retract the piston holder and can stop the piston attachment at the desired position by long-pressing the printer knob, otherwise it should turn off automatically when the limit switch is triggered.
5. The wheel is then turned manually until the syringe piston-holder slides into place.
6. The Arduino should be in ready mode after loading (status LED green). If not, switch to the ready mode by using the change button

After either loading method the securing screws should be tightened (Fig. 11).

Other variations of the loading file can be created depending on preference. Examples are attached, including one that moves backward and one that moves in steps and then waits for user input.

### 6.4 Printing

Printing with the Gelstruders is similar to printing with the plastic. However, it is a good idea to first run the print without any gel but with a syringe tip in place, to ensure no mistakes occurred when slicing the model with the custom G-codes.

The printing routine is as follow.

1. Setup the printing surface: steel sheet, Petri dish, etc.
2. Start the print via the “Print from the SD” menu. (The status LED should now turn to the printing mode. If not, change the printing mode by pressing the Change button.)
3. Monitor the progress while printing. Adjustments can still be done by accessing the “Live-tune” LCD menu as with the plastic printing.
4. At the end of the print make sure that the printer is in “ready mode” (Green status LED), whether it finished normally or was aborted due to a lack of gel.

If printing with 2 extruders, the user should keep in mind that at the start of every print job, the Gelstruder 1 is selected by default. Additionally, some material needs to be ejected in order to make

## 7 Validation and characterization

The printer maintains the same spatial resolution and nearly the same theoretical printing volume as the original Prusa printer, and is summed up in Table 3. The system has a high precision tolerance, yet in practice the resolution and maximum print size depends on the size of the syringe tip used as well as the bio-ink used. There is a trade off between the rigidity of the hydrogel (i.e. increasing the viscosity) and the pressure build-up while printing. Hydrogels tend to sag over time (before curing); therefore, the z height is limited by the rigidity of the bio-ink. This might be overcome by using UV curing between layers, however this in turn might result in killing the cells in the lower layers due to overexposure to UV. A smaller tip can also be used for finer print, yet this in turn increases the pressure in the system.

**Table 3:** Printer theoretical bed volume and resolution.

Parameter	Set value
Max bed volume (X,Y,Z)	230 × 200 × 100 mm
Theoretical precision tolerance	0,1 mm in Z and 0,3 mm in X&Y
Physical layer height	Depends on tip size and material (1 mm for 18G nozzle)
Layer width	Depends on tip size (1 mm for 18G nozzle)
Minimum print size	Depends on tip size and material (approx. 1 mmx 2 mm for 18G nozzle)

**Fig. 12:**
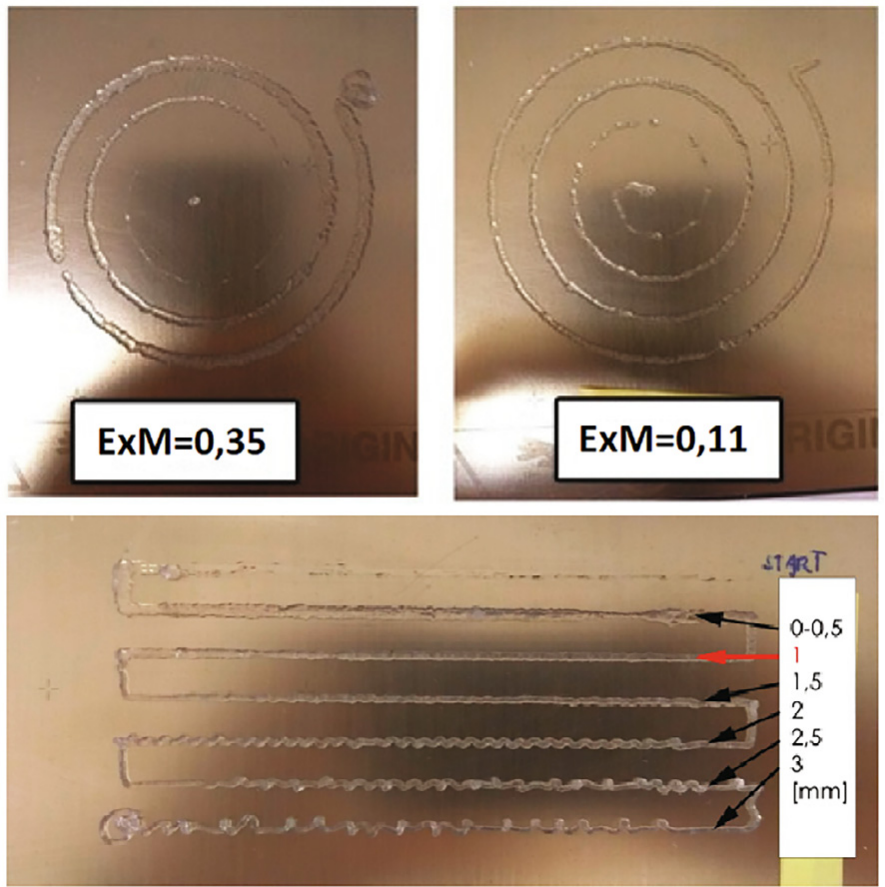
Flow rate and layer-height parameter test.

For the testing purposes, a biocompatible, hyaluronic acid based hydrogel (2% each of sodium hyaluronate, k-carrageenan and fumed silica, [11]) was chosen. This is a relatively viscous or rigid ink, and therefore a relatively large syringe size was used (18G) to minimize the pressure build-up in the system. Various printing parameters were tested and adjusted accordingly. The parameters were optimized using simple one layer prints Fig. 12. The tested parameters and their optimized values are shown in Table 4.

**Table 4:** Optimized parameter values.

Parameter	Set value
Print speed	20 mm/s
Extrusion multiplier	0,11
layer height (18G nozzle)	1 mm

**Fig. 13:**
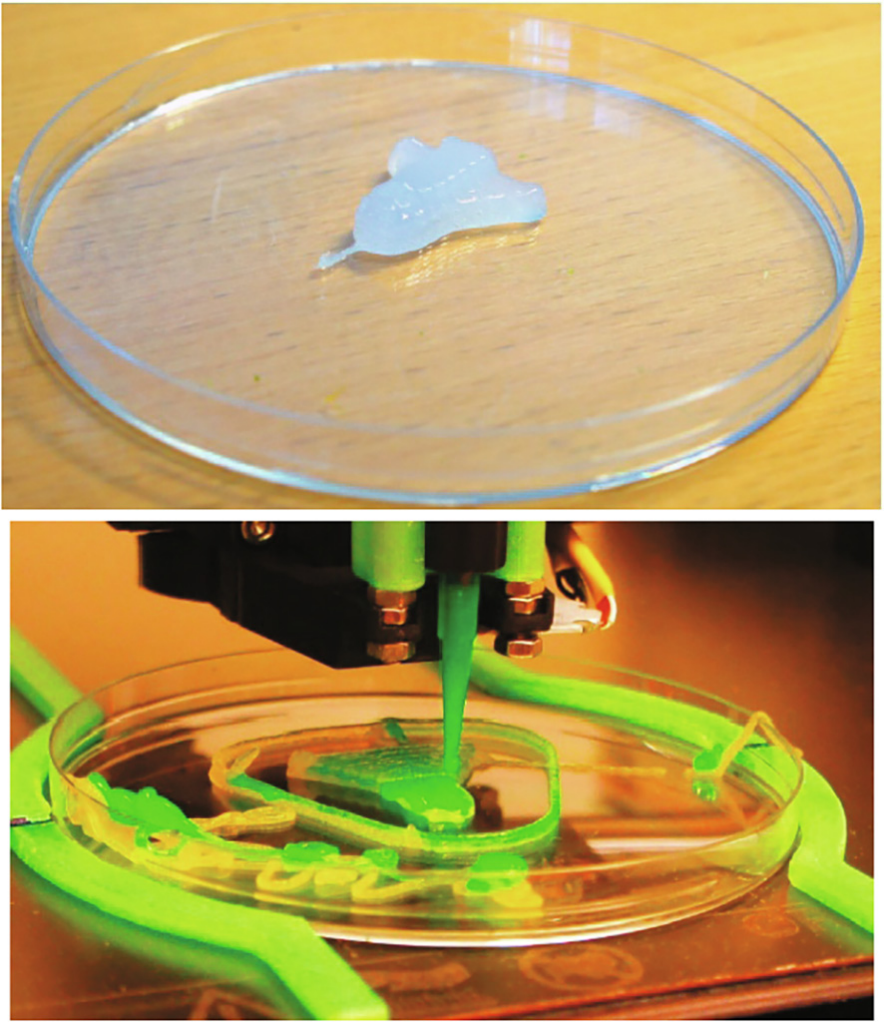
Dual material print in process.

After various tests to find the correct parameters, test prints were done using both a single gel and two gels, Fig. 13. A small rocket was chosen as a test model.

### 7.1 Bio-printing validation

**Fig. 14:**
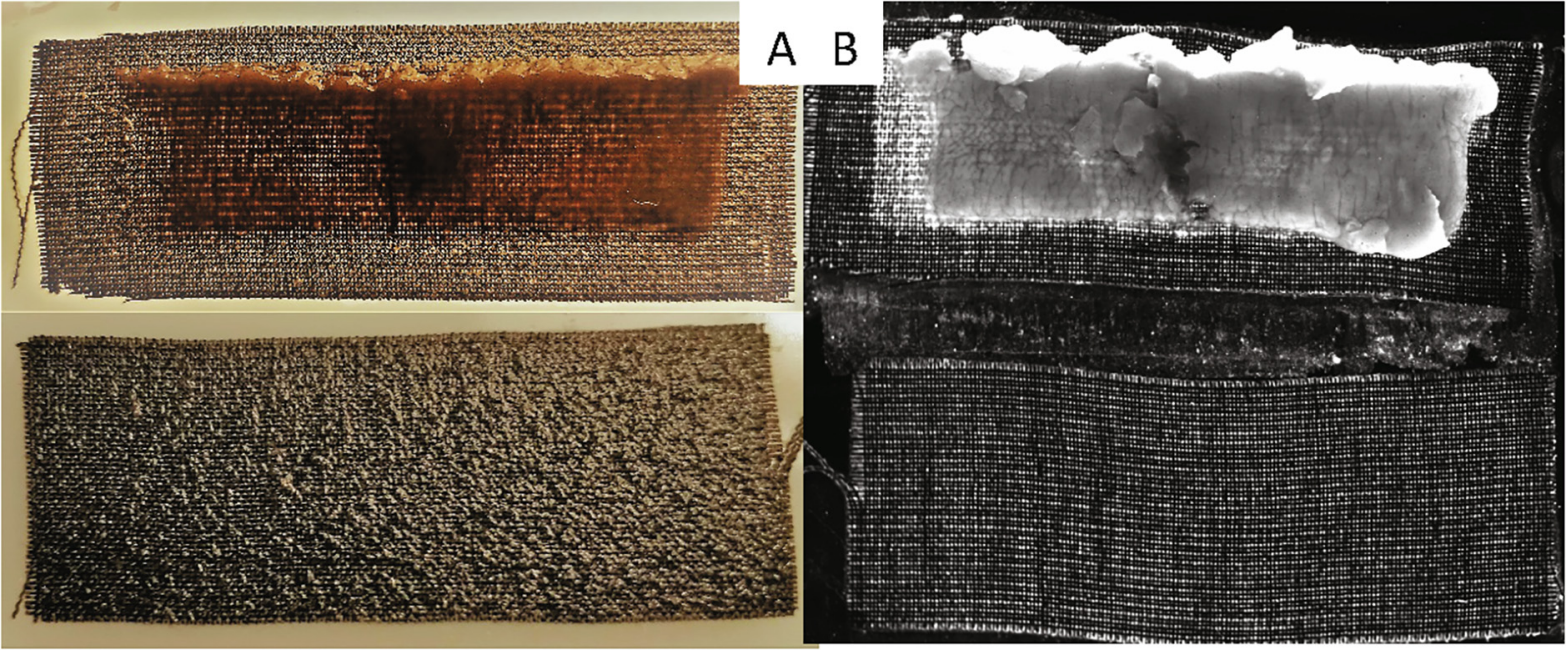
A) Image of the printed biofilm electrode (top) and a control (bottom) after 40 h in a microbial fuel cell B) the same electrodes under UV after staining and soaking in distilled water.

To test the validity of the bioprinter for cell-laden hydrogels, a commercially available UV-curing hydrogel (GelMa, Cellink) was used to print the electro-active bacteria, *Geobacter sulfurreducens* onto a carbon cloth electrode. The electrode was then cured using the UV-LED (15 s curing time) and placed in a standard H-type microbial fuel cell, which generates electricity via direct electron transfer by consuming acetate. Details on the media and strain used can be found in Krige et al. [12]. The reactor was maintained for approximately 40 h before the printed electrode was washed in distilled water and stained with a UV-fluorescent stain to show the cell retention, seen in Fig. 14. The red colour in Fig. 14 A is caused by the large number of cytochromes in the cells. A control microbial fuel cell was also used which was inoculated with a suspended culture. This shows the high cell retention capabilities of the 3D-printed biofilm, when compared to a suspended culture, as well as the ability to effectively cure a hydrogel using the UV LED without killing the cells. Typical biofilms using suspended cultures take approximately a week to grow to a point where the colour is that evident.

**Fig. 15:**
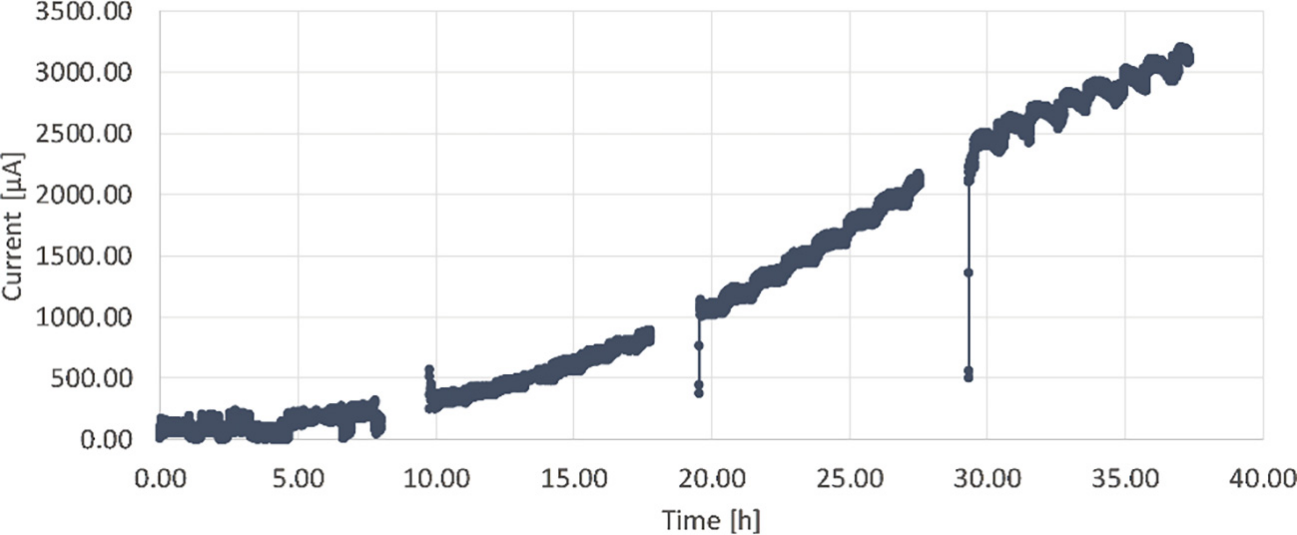
Current produced using a 3D-printed synthetic biofilm.

The growth of the cells can also be directly observed via the current production in the microbial fuel cell (Fig. 15).

In conclusion, the printer kept the properties of the original 3D printer. Therefore, the accuracy of the X and Y axis is the same as at the original one. In Z axis the accuracy depends on the size of the tip used (about 1 mm using a 18G nozzle). Finally, taking into consideration that the gel has tendency to smear or sag a bit, the overall accuracy of the print can be somewhere in 0,5–1 mm range depending at the gel viscosity. It is also capable of printing and curing cell-laden hydrogels that maintain its structure for several days in a bio-reactor.

This printer works best when printing simple structures, where each layer can be printed in one line without stopping and hopping to other point.

When using the dual printing function, one has to take into consideration the waste of material while purging out the nozzle. Using one nozzle works best for applications where it does not matter that the material mix together somewhat. It is not suitable for reactive materials such as an epoxy or similar.

## 8 Human and animal rights

No humans or animals were used in the research.

## Declaration of Competing Interest

This research was funded by the 10.13039/501100004359Swedish Research Council (VR), project number 2018**-**03875.
